# The structure and diversity of bacteria and fungi in the roots and rhizosphere soil of three different species of *Geodorum*

**DOI:** 10.1186/s12864-024-10143-2

**Published:** 2024-02-28

**Authors:** Jianxiu Liu, Danjuan Zeng, Yang Huang, Lisha Zhong, Jialin Liao, Yuxing Shi, Haidu Jiang, Yajin Luo, Yu Liang, Shengfeng Chai

**Affiliations:** 1 Key Laboratory of Ecology of Rare and Endangered Species and Environmental Protection, Guangxi Key Laboratory of Landscape Resources Conservation and Sustainable Utilization in Lijiang River Basin, College of Life Science, Guangxi Normal University, Guilin, China; 2 Guangxi Key Laboratory of Plant Functional Phytochemicals and Sustainable Utilization, Guangxi Institute of Botany, the Chinese Academy of Sciences, Guilin, China; 3 School of Mechanical and Electrical Engineering, Guilin University of Electronic Technology, Guilin, China; 4 Yachang Orchid National Nature Reserve Management Center, Baise, China

**Keywords:** *Geodorum*, Orchidaceae, Roots, rhizosphere soil, Bacteria, Fungi

## Abstract

Shepherd's crook (*Geodorum*) is a genus of protected orchids that are valuable both medicinally and ornamentally. *Geodorum eulophioides* (GE) is an endangered and narrowly distributed species, and *Geodorum densiflorum* (GD) and *Geodorum attenuatum* (GA) are widespread species. The growth of orchids depend on microorganisms. However, there are few studies on the microbial structure in *Geodorum*, and little is known about the roles of microorganisms in the endangered mechanism of *G. eulophioides*. This study analyzed the structure and composition of bacterial and fungal communities in the roots and rhizosphere soil of GE, GD, and GA. The results showed that *Delftia*, *Bordetella* and norank_f_Xanthobacteraceae were the dominant bacteria in the roots of *Geodorum*, while norank_f_Xanthobacteraceae, *Gaiella* and norank_f_norank_o_Gaiellales were the dominant bacteria in the rhizosphere soil of *Geodorum*. In the roots, the proportion of *Mycobacterium* in GD_roadside was higher than that in GD_understory, on the contrary, the proportion of *Fusarium*, *Delftia* and *Bordetella* in GD_roadside was lower than that in GD_understory. Compared with the GD_understory, the roots of GD_roadside had lower microbial diversity. In the endangered species GE, *Russula* was the primary fungus in the roots and rhizosphere soil, with fungal diversity lower than in the more widespread species. Among the widespread species, the dominant fungal genera in the roots and rhizosphere soil were *Neocosmospora*, *Fusarium* and *Coprinopsis*. This study enhances our understanding of microbial composition and diversity, providing fundamental information for future research on microbial contributions to plant growth and ecosystem function in *Geodorum*.

**Supplementary Information:**

The online version contains supplementary material available at 10.1186/s12864-024-10143-2.

## Introduction

Shepherds' crooks (*Geodorum*), a genus of the Orchidaceae, has been listed as protected plants in China. *Geodorum* includes approximately ten species. There are five species of *Geodorum* in China, namely *G. densiflorum* (GD), *G. recurvum*, *G. pulchellum*, *G. attenuatum* (GA) and *G. eulophioides* (GE) [1]. *Geodorum* is a type of plant that is protected at a national level due to its ornamental and medicinal value. It was listed in Appendix II of the Convention on International Trade in Endangered Species of Wild Fauna and Flora (CITES), and GE was listed in the International Union for Conservation of Nature (ICUN) Red List of Endangered Species. The growth and development of plants are closely linked to soil microorganisms. Analyzing the composition of soil microorganisms can aid in promoting plant protection [2]. GE is highly valuable ornamentally, but it has a narrow area of distribution [3]. In contrast, the other species of *Geodorum* are distributed over a wide area. GD is the most widespread and abundant speciess within *Geodorum*. GD predominantly grows in areas at 1,500 m altitude, including sparse forests, roadsides, and grassy slopes, and is found in South and Southwest China, Vietnam, Laos, Cambodia, Thailand, Malaysia, and Indonesia, among other countries. GA primarily grows in the forest margin and sparse forest areas below an altitude of 800 m. It is distributed in Hainan, Yunnan and the southern Guangxi Province of China, and Vietnam, Laos and Myanmar. The range of distribution of GA is smaller than that of GD. GE is only found at the junction of Yunnan, Guizhou and Guangxi Provinces in China, and it grows at an altitude of 600 m in shrubs or medium shade forests in limited quantities; it is very narrowly distributed and found in limited quantities [1]. Since orchids thrive in specific habitats, the microorganisms in their rhizosphere soil play a crucial role in adaptation, while the soil environment influences the survival of these microorganisms [4]. For instance, orchid species growing in mining areas often contain various toxic elements. Based on 16S rRNA gene and ITS amplification and sequencing of the roots and soil of narrow-leaved helleborine (*Cephalanthera longgifolia*), *Epipactis pontica*, royal helleborine (*E. atrorubens*), and lesser-butterfly orchid (*Platanthera bifoli*), no significant difference in microbial composition was found among the orchids from different mining areas, and bacteria and fungi could reduce the damage of toxic elements to orchids [5]. In the same way, the survival and reproduction of orchids require a specific environment, and they generally grow in low altitude areas, primarily in the understory, grasses, bushes, and roadsides [1]. Like other orchids, the germination of *Geodorum* seeds depends on the mycorrhizal fungi associated with their roots [6]. Mycorrhizal fungi are not only essential for the germination of orchid seeds but also assist in the uptake of nutrients by their roots to promote their growth and reproduction [7]. Therefore, understanding the fungal and bacterial composition in orchids' natural environments is essential for conservation efforts of these valuable plants.

Currently, most species of orchids have been listed as key protected wild plants, particularly species of *Cymbidium*, *Dendrobium* and *Paphiopedilum*. Therefore, strengthening the protection of wild orchids is imperatives. Many studies have focused on various aspects of orchid reproduction, including genetic diversity [8–10], reproductive techniques [11] and mycorrhizal fungi [12–14]. Microorganisms in the plant rhizosphere soil and roots affect the decomposition of soil organic matter and the absorption of nutrients by plant roots, and some harmful bacteria can also cause plant diseases. Studying the characteristics of bacteria and fungi in orchid roots and rhizosphere soil can reveal how these microorganisms affect orchid growth and distribution across habitats. Based on an analysis of microbial composition and diversity in the rhizosphere soil of *Holopogon pekinensis* in different regions, the dominant bacteria and microbial community richness of *H. pekinensis* were found to be related to the species of trees in its habitat [15]. The composition and diversity of bacteria and fungi in different tissues and other aspects of various orchids have been studied to analyze the interaction between orchids and microbial communities and to provide reliable guidance for their cultivation [16–19].

*Geodorum* is an herbaceous plant in the family Orchidaceae. The studies of this genus focus on its genetic diversity and analyses of its embryology [20–23]. There has been very little research on the effect of mycorrhizal fungi on the germination and growth of *Geodorum* seeds. Therefore, as an endangered species, the fungal composition in the root and rhizosphere soil of GE may be related to its endangered status. The reasons for its precarious status were analyzed in terms of human factors, the morphology of GE, and population competition [3]. To date, to our knowledge, there have been no relevant studies on the link between microbial diversity in the root and rhizosphere soils of *Geodorum* and causes of its endangered status. There were two types of habitats for GD. One included the understory (GD_understory), while the other included roadsides (GD_roadside). However, it is not clear whether there are differences in the microbial composition of GD_understory and GD_roadside and how the rhizosphere microorganisms affect their growth. The growth of *Geodorum* was influenced by various factors such as soil properties and altitude, subsequently impacting its interactions with soil microorganisms. Additionally, the species' distribution range was also influenced by its specificity to certain fungi. A study on the diversity of bacteria and fungi in the roots and rhizosphere soil of *Geodorum* in different habitats could provide insights into the interactions between microorganisms and this genus of orchids.

In this study, the roots and rhizosphere soil of three species of *Geodorum* (GE, GD and GA) were studied. The bacterial and fungal diversity was investigated by 16S rRNA and ITS amplification and the sequencing of root and rhizosphere soil samples from *Geodorum*. Differences in the bacterial and fungal diversity in the root and rhizosphere soil between different habitats and endangered species (GE) and widespread species (GD and GA) should help to identify possible relationships between the microbial diversity and endangered species and the relationship between microorganisms and the habitats of species of *Geodorum*.

## Materials and methods

### Plant roots and soil sampling

**Fig. 1:**
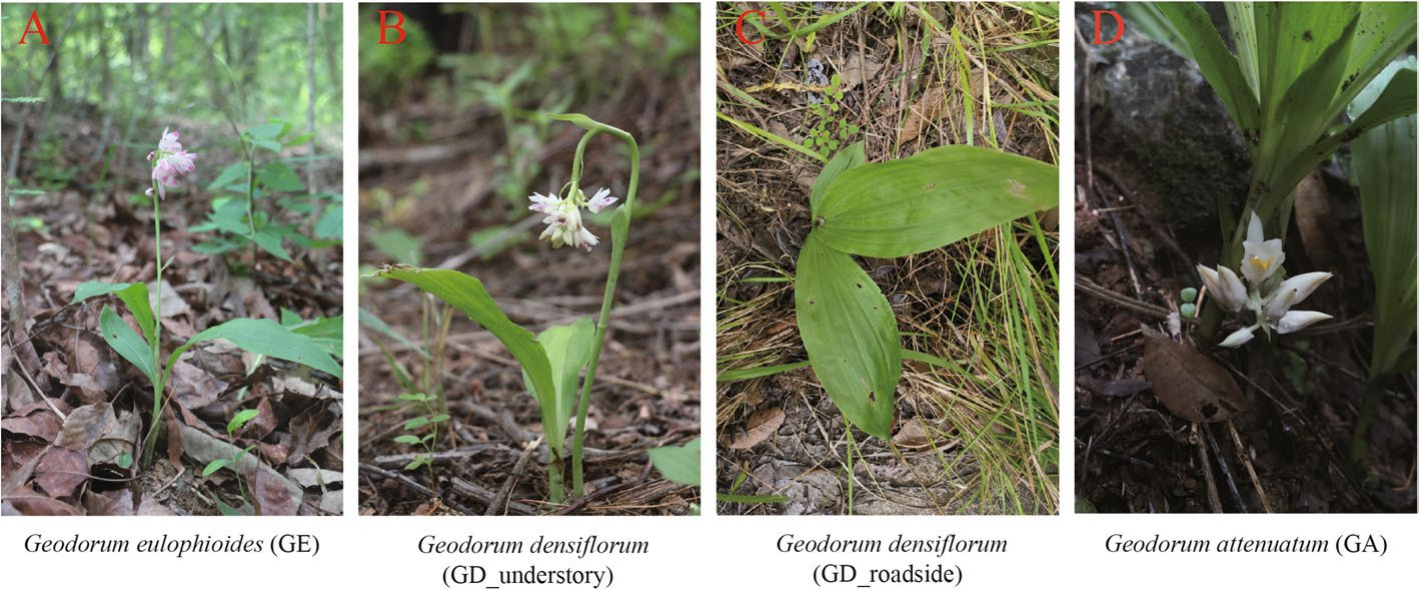
Photographs of the habitat of *Geodorum*. **A**: *Geodorum eulophioides*; **B** and **C**: *Geodorum densiflorum* (**B**: growing in the understory; **C**: growing in the roadside); **D**: *Geodorum attenuatum*

Roots and rhizosphere soils were collected from three species of *Geodorum* in China. GE, GD, and GA were collected from the Yachang Orchid Nature Reserve, Baise City, Guangxi Province, and Minqiang Village, Longzhou County, Chongzuo City, Guangxi Province, China. In addition, GD was sampled from understory (GD_understory) and roadside (GD_roadside) habitats. GE and GA were both sampled from the understorey. While GE was sampled in Baise City, GA was sampled in Chongzuo City. In addition, they grew in different locations at varying altitudes. The sampling site information of *Geodorum* was shown in Table 1, and the habitat picture was shown in Fig. 1.

**Table 1:** Sampling information of three species of *Geodorum*

Species	Altitude (m)	Habitat	Sampling location
*Geodorum eulophioides*	525	Understory	Xiaya Small-protected-area, Yachang Orchid Nature Reserve, Leye county, Baise city, Guangxi (24°57′3″ N, 106°9′2″ E)
*Geodorum densiflorum*	500	Roadside	Xiaya Small-protected-area, Yachang Orchid Nature Reserve, Leye county, Baise city, Guangxi (24°57′3″ N, 106°9′2″ E)
*Geodorum densiflorum*	445	Understory	Ergou district, Yachang Orchid Nature Reserve, Leye county, Baise city, Guangxi (24°47′4" N, 106°12′25" E)
*Geodorum attenuatum*	294	Understory	Minqiang Village, Longzhou County, Chongzuo City, Guangxi (22°25′14"N, 106°54′17" E)

The sampling sites of *Geodorum* were all low-altitude areas. The sampling sites of GE have a dry climate (annual average rainfall was 1216.9–940.8 mm) and annual average temperature was 19.2–20.4 °C, and especially the growth soil type was red soil. In addition, there were trees in the upper layer of habitat of GE, including *Pinus yunnanensis*, and grass in the shrub layer, such as *Phyllodium pulchellum*, *Callicarpa bodinieri* and *Chromolaena odorata*. However, the habitat of GD_roadside lacked an upper layer of trees and was situated at an altitude of 500 m. GD_roadside thrives in full light and the soil was of a sandy composition, with an environment characterized by an admixture of gravel and soft soil. Unlike the habitat of GD_roadside, the habitat of GD_understory was characterized by its upper tree layers, which include species such as *Vernicia fordii*. GA grew on the periphery of wooded areas, shunning direct sunlight, in mildly acidic and soft soil, with an annual average temperature of 20–28 °C in Chongzuo City.

Three plants from GA, GD_understory, GD_roadside, and GE distributions were randomly selected as biological replicates. Three biological replicates were used for both the root and rhizosphere soil samples, and they yielded 24 samples in total. After the non-rhizosphere soil of the plants was removed by shaking, about 5 g of soil about 3 mm away from the roots was collected as rhizosphere soil samples. Roots were washed with sterile water to remove soil before sample collection. Five grams of rhizosphere soil per plant and over 20 root fragments per plant were collected after removing the rhizosphere soil. Samples were collected and stored on in -80ºC and then used for 16S rRNA and ITS sequencing.

### Measurement of soil properties at sampling sites

Approximately 50 g of soil samples were collected at each sampling site and airdried outdoors for 5 days after collection. Soil pH was measured using a PHS-3C acidity meter (Shanghai INESA, Shanghai, China). Weigh 10 g of air-dried soil sample in a 50 ml beaker, add 25 ml of distilled water, stir and mix well, and let stand for 30 min. The above to be tested solution was determined, each sample was repeated three times and the average value was taken.

Soil organic matter was determined using the potassium dichromate volumetric method [24]. 0.1 g of soil sample through 60 mesh sieve was weighed into a test tube, and 10 ml of 0.136 mol/L K_2_Cr_2_O_7_-H_2_SO_4_ solution was added to an oil bath at 170 ºC for 5 min. After cooling, it was transferred to a 250 ml triangle flask with distilled water, and 3 drops of 1,10-Phenanthroline indicator (200–629-2, Shanghai, China) was added. The solution was titrated with 0.2 mol/L FeSO_4_ solution to change from yellow to green to brown–red. Quartz sand was used as blank control. The soil organic matter content was calculated according to the formula (V_0_: The volume of FeSO_4_ used to titrate the blank solution. V: The volume of FeSO_4_ used to titrate the sample solution. N: concentration of standard FeSO_4_.):

$$Organic\;matter\;content\left(\frac g{kg}\right)=\frac{\left[\left(V_0-V\right)N\ast0.003\ast1.724\ast1.1\right]\ast1000}m$$

The available nitrogen in soil was determined by potassium dichromate and sulfuric acid digestion method [24]. After air drying, 0.5 g of soil sample was weighed and placed into a 150 ml digestion tube through 60 mesh sieve. Then, 5 ml of H_2_SO_4_ was added and boiled on a digestion furnace at high temperature for 20 min. After cooling, 5 ml of K_2_Cr_2_O_7_ solution was added and heated on an electric furnace for 5 min. The above solution was then distilled for about 20 min to complete distillation. Steam was passed through a condensing tube into a triangular bottle containing 25 ml of 2% boric acid absorption solution and 1 drop of nitrogen-mixed indicator (Thermo Scientific, MA, USA). The resulting solution was titrated with a 0.02 mol/L standard solution of hydrochloric acid. The solution changed from blue to burgundy, and the volume of hydrochloric acid used was recorded. The available nitrogen content in soil samples was calculated by this formula(V_0_: The volume of hydrochloric acid used to titrate the blank solution. V: The volume of hydrochloric acid used to titrate the sample solution. N: concentration of standard hydrochloric acid.):

$$Available\;nitrogen\;content\left(\frac{mg}{kg}\right)=\frac{\text{N}\ast\left(V-V_0\right)\ast14\ast1000}m$$

The available phosphorus in soil samples was determined by sodium bicarbonate method [25]. 5 g of airdried soil samples through 18 mesh sieve were weighed into a triangular flask, 0.1 g of phosphate-free active carbon was added, and the mixture was shaken for 30 min. The filtrate was filtered and placed in triangular flask. Take 10 ml of filtrate in a 50 ml volumetric flask, add 2 drops of dinitrophenol indicator (Thermo Scientific, MA, USA), add 5 ml of molybdenum antimonium sulfate mixed color developing agent (Thermo Scientific, MA, USA) and shake thoroughly, discharge carbon dioxide and add water to scale, and then shake thoroughly. After 30 min, a spectrophotometer (Thermo Scientific, MA, USA) was used to measure the value at 600 nm, and a standard curve was drawn and the available phosphorus content in the soil was calculated.

Weigh a 0.5 g soil sample and pass it through an 18 mesh sieve into a triangular flask. Add 50 ml of 1 mol/L NH_4_OAc solution and shake the mixture at 20–25 ºC for 30 min. Filter the mixture through dry filter paper and use a flame photometer (FP6430, Shanghai, China) to measure the available potassium content in the soil. Draw a standard curve and calculate the available potassium content in the soil.

### DNA extraction, PCR, and sequencing

Genomic DNA was extracted using an E.Z.N.A.® Soil DNA Kit (Omega Bio-tek, sNorcross, GA, USA). A NanoDrop2000 spectrophotometer (Thermo Fisher Scientific, Waltham, MA, USA) was used to determine the purity and concentration of genomic DNA, and the DNA integrity was detected by 1% agarose gel electrophoresis at 5 V/cm for 20 min. For PCR amplification, primers 515F (5'—barcode—GTGCCAGCMGCCGCGGTAA—3')/ 806R (5' – GGACTACHVGGGTWTCTAAT—3') were used to amplify the V3—V4 region of 16S rRNA genes in the rhizosphere soil samples, and primers 799F (5'—barcode – AACMGGATTAGATACCCKG—3')/1193R (5' – ACGTCATCCCCACCTTCC—3') were used to amplify the V5—V7 region of 16S rRNA genes in the root samples. In addition, ITS1F (5 ' -barcode—CTTGGTCATTTAGAGGAAGTAA—3')/ITS2R (5'—GCTGCGTTCTTCATCGATGC—3') primers were used to amplify the ITS1 gene region of fungi [26]. The PCR reaction system contained 20 μL in total, with 10 ng of DNA template, 0.5 μM primer, 0.4μL FastPfu DNA polymerase (TransGen, China), 2μL 2.5 mM dNTPs, 4μL 5 × FastPfu Buffer, and 0.2μL BSA. The PCR reaction followed these conditions: 3 min denaturation at 95 °C, 30 cycles (30 s of denaturation at 95 °C, 30 s of annealing at 56 °C, 1 min of extension at 72 °C), and a final extension step of 10 min at 72 °C. The PCR products were then identified, purified, and quantified. The PCR products were identified by electrophoresis on a 2% agarose gel, purified with an Axygen Biosciences Gel Exact kit (Axygen Biosciences, Union City, CA, USA), and quantified with a Quantus™ Fluorometer (Promega, Madison, WI, USA). According to the sequencing volume requirements of each sample, the corresponding proportions were mixed. After MiSeq library construction, sequencing was performed on a MiSeq PE300 platform (Illumina, San Diego, CA, USA).

### Sequence data analysis

The raw data were obtained after Illumina sequencing. FASTP (https://github.com/OpenGene/fastp, version 0.20.0) was used to remove low-quality sequences [27], and FLASH (http://www.cbcb.umd.edu/software/flash, version 1.2.7) was used to assemble the sequences [28]. During raw data quality control, bases with quality values < 20, reads shorter than 50 bp and reads containing N bases were removed. Sequence splicing was used to merge pairs of paired-end (PE) reads into a single sequence based on the overlapping relationship between the PE reads, and the minimum overlap length was 10 bp.

### Statistical analysis

Data analyses were conducted using the MegBio Cloud platform (https://cloud.majorbio.com).

#### OTU analysis

Operational taxonomic unit (OTU) clustering to identify species and quantify bacteria and fungi in samples was performed using UPARSE software (version 7.1, http://drive5.com/uparse/) [29]. All the optimized sequences were mapped to the OTU representative sequences, and sequences > 97% similar to the OTU representative sequences were selected to generate the OTU table (Table S2, S3). The OTUs of bacteria and fungi were compared with the Silva 16S rRNA gene database (Release138 http://www.arb-silva.de) and UNITE 8.0/ITS_fungus database (Release 8.0 http://unite.ut.ee/index.php) to annotate the classification of species by OTU, respectively [30]. Rarefaction curve analysis showed Good's coverage for observed OTUs in all samples > 97%, indicating sufficient sequencing depth for subsequent analyses (Fig. S1).

#### Venn diagram

During the analysis, the raw data were first subjected to the steps of quality control, clustering, and assignment of taxa, resulting in an OTU table for each sample. Next, use R (version 3.3.1) draw Venn diagram [31].

#### Community Composition Analysis: Bar Chart

The OTU table obtained after pre-processing the raw data was used to draw the community bar chart using R (version 3.3.1). The data were converted by percentage of relative abundance, and then the data were grouped according to the taxonomic level to draw the stacking bar chart.

#### α-Diversity analysis: Index group difference test

The Chao index is commonly used to estimate the total number of species, and the Shannon index is often used to reflect the diversity of species, with a higher Shannon value indicating a higher community diversity. The OTU diversity index table was first obtained by pre-processing the raw data, namely the OTU analysis described above. The choice was then made to calculate a diversity value for each sample using the Chao index, reflecting the richness and evenness of the microbes in the sample. Differences in diversity between groups were tested using the Kruskal–Wallis rank sum test to assess whether there were significant differences in diversity indices between samples (GE, GD_understory, GD_roadside and GA) [32]. The diversity differences were visualized using box plots in R (version 3.3.1).

#### β-Diversity analysis: NMDS analysis

A non-metric multidimensional scaling (NMDS) analysis was conducted using the Bray–Curtis distance algorithm to represent the multidimensional space as points. The difference between different samples is reflected by the distance between points, and the spatial anchor map of the samples was finally obtained. The OTU table was obtained after pre-processing the raw data, and NMDS ordination was used to reduce the dimension and visualise the similarity between samples by performing NMDS analysis based on the distance matrix. In the comparison of differences between multiple groups, multiple comparisons were corrected by false discovery rate (FDR) [33]. Finally, according to the results of difference analysis, data were visualized using scatter diagram through R (version 3.3.1).

#### ANOSIM analysis

Analysis of similarities (ANOSIM) was used to test whether the differences between groups (two or more groups) were significantly greater than the differences within groups. The distance between pairs of samples was calculated using the distance algorithm (Bray–Curtis), and data were visualized using box plots through R (version 3.3.1).

#### Species difference analysis

After preprocessing the raw data, the OTU table was used for species differentiation analysis, and differences between groups were evaluated using the Kruskal–Wallis rank sum test, with statistical significance set at *P* < 0.05. In the comparison of differences between multiple groups, multiple comparisons were corrected by false discovery rate (FDR) (FDR < 0.05). Finally, according to the results of difference analysis, data were visualized using bar charts through R (version 3.3.1).

#### Co-occurrence network analysis

Co-occurrence network analysis can be used to show the distribution between samples and species. By analyzing the species abundance information among different samples, the co-occurrence relationship of species in environmental samples can be obtained, which can highlight the similarities and differences between samples. Associations with Reads Per Kilobase Million (RPKM) ≥ 200, a *P*-value < 0.05 and an *R*-value > 0.6 were retained in the network. Co-occurrence network analysis was performed according to the OTU table after preprocessing of the raw data. The co-occurrence network analysis of species abundance information between different samples was performed using Networkx (vsesion1.11), and the abundance of different microbial species in each sample was calculated. Based on the RPKM abundance data of microbial species, a co-occurrence matrix was constructed and rare species were filtered. spearman correlation coefficient was used to calculate the symbiosis between microbial species, and significant co-occurrence associations were transformed into co-occurrence network maps according to the *P*-value and *R*-value.

##### Multilevel species discriminant analysis: LEfSe analysis

Linear discriminant analysis Effect Size (LEfSe) combined linear discriminant analysis (LDA) and measures of effect size to identify microbial signatures with significant differences. Analysis using LEfSe software (http://huttenhower.sph.harvard.edu/galaxy/root?tool_id=lefse_upload), LDA and effect size measure (the LDA threshold was 3) found significant differences in microbial characteristics. In LEfSe, Kruskal–Wallis test was used to identify microorganisms with significant differences, and LDA analysis was used to determine the impact of these differences on sample grouping.

##### RDA/CCA analysis

Redundancy Analysis (RDA) and Canonical Correspondence Analysis (CCA) were commonly used to explore the effects of environmental factors on species composition. According to the selection principle of RDA or CCA model, DCA analysis was performed with the OTU table with 97% similarity. When the first axis of Lengths of gradients in the analysis results was greater than 3.5, CCA was selected, and the Lengths of gradients were less than 3.5, RDA was selected. The R language vegan(vsesion2.4.3) was used for CCA or RDA analysis and mapping.

## Results

### Soil information at sampling sites for the three species of *Geodorum*

Soil property information, including soil pH, organic matter, available nitrogen, available phosphorus, and available potassium, was analyzed at the four sampling sites. Soil properties regarding sampling sites were shown in Table S1. GE and GA soils tended to be acidic (pH < 7) while GD_understory and GD_roadside soils tended to be alkaline (pH > 7). The content of soil organic matter content and available nitrogen in GE and GD_roadside was significantly lower than that in GD_understory and GA. The content of available potassium in GE and GD_roadside soils was significantly higher than that in GD_understory and GA. The results of RDA and CCA showed that pH, altitude, available nitrogen and organic matter were significantly correlated with the composition of the microbial community (Figure S2). The community composition of bacteria and fungi in rhizosphere soil and roots of GA was mainly correlated with altitude, available nitrogen and organic matter content. Moreover, pH was negatively correlated with available nitrogen and organic matter in the soil. Organic matter and available nitrogen in the soil were mainly correlated with the fungal composition in the roots and rhizosphere soils of GD_roadside and GD_understory(Fig. S2B).

### Bacterial composition of three species of *Geodorum* that grow in understory and roadside areas.

**Fig. 2:**
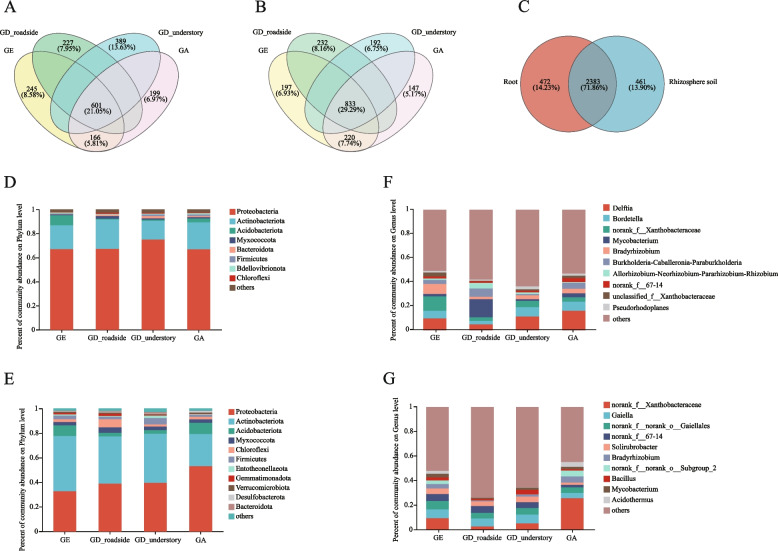
Bacterial composition of different *Geodorum* species. **A**, **B** and **C**: Venn diagram of different groups at the OTU level (A: in the roots of GE, GD_roadside, GD_understory and GA groups; B: in the rhizosphere soil of GE, GD_roadside, GD_understory and GA groups; C: in the root and rhizosphere soil groups in all the *Geodorum* species). **D** and **E**: Bar graphs of the bacterial community composition at the phylum level (D: in the roots of *Geodorum*; E: in the rhizosphere soil of *Geodorum*). F and G: Bar graphs of the bacterial community composition at the genus level (F: in the roots of *Geodorum*; **G**: in the rhizosphere soil of *Geodorum*)

A total of 1,228,959 sequence numbers were obtained with an average length of 377 base pairs (bp), the shortest sequence after 16S rRNA sequencing was 200 bp, and the longest sequence was 520 bp (Table S4). A taxonomic analysis was performed on the 97% similarity level of the OTU representative sequences, and the community species composition of each sample was then calculated. At the OTU taxonomic level, the number of OTUs for bacteria in the rhizosphere soil was higher than that in the roots of all *Geodorum* (Table 2). *Geodorum* roots shared 601 (21.05%) common 16S OTUs, with GD_understory having the highest number of unique OTUs at 389 (13.63%) (Fig. 2A). However, a total of 833 (29.29%) common 16S OTUs were found in the rhizosphere soil of *Geodorum*, which was higher than that in the roots, indicating that the difference of bacterial species in the roots of different *Geodorum* was greater than that in the rhizosphere soil (Fig. 2B). A total of 2,383 (71.86%) 16S OTUs were common between bacteria in the rhizosphere soil and roots, with 472 (14.23%) and 461 (13.90%) unique OTUs in each, respectively (Fig. 2C).

**Table 2:** The information on 16S OTU classification statistics in *Geodorum*

Sample	Domain	Kingdom	Phylum	Class	Order	Family	Genus	Species	OTU
GE_S	1	1	25 ± 0ab	60 ± 2ab	129 ± 4bc	202 ± 6ab	342 ± 7abc	623 ± 20ab	1276 ± 39a
GE_R	1	1	23 ± 3b	46 ± 7c	107 ± 9d	168 ± 13c	263 ± 34d	439 ± 87d	796 ± 198c
GD_roadside_S	1	1	26 ± 1a	63 ± 1a	140 ± 3abc	214 ± 2ab	364 ± 7ab	642 ± 16ab	1257 ± 50a
GD_roadside_R	1	1	25 ± 2ab	53 ± 6bc	123 ± 13 cd	188 ± 21bc	295 ± 26 cd	478 ± 62 cd	800 ± 166c
GD_understory_S	1	1	27 ± 1a	63 ± 1a	149 ± 3a	228 ± 6a	384 ± 14a	690 ± 32a	1316 ± 58a
GD_understory_R	1	1	28 ± 1a	62 ± 6ab	146 ± 3ab	226 ± 14a	32ab	665 ± 54ab	1164 ± 145ab
GA_S	1	1	25 ± 1ab	55 ± 2abc	126 ± 1c	198 ± 2b	334 ± 5bc	603 ± 9ab	1145 ± 42ab
GA_R	1	1	25 ± 1ab	57 ± 3ab	133 ± 7abc	211 ± 12ab	338 ± 19abc	568 ± 19bc	960 ± 14bc

*OTU* operational taxonomic unit

The bacterial community composition of different species of *Geodorum* in the roots and rhizosphere soil varied. At the phylum level, Proteobacteria, Actinobacteriota, Acidobaceriota, Myxococcota and Bacteroidota were the top 5 most abundant bacteria in the roots (Fig. 2D). In addition, Proteobacteria, Actinobacteriota, Acidobaceriota, Myxococcota and Chloroflexi were the top 5 most abundant bacteria in the rhizosphere soil (Fig. 2E). The difference was that the proportion of Actinobacteriota in the rhizosphere soil (26%—45%) was higher than that in the roots (15%—24%) (Fig. 2D, E).

The composition and proportion of bacterial communities at the genus level in the roots and rhizosphere soil of *Geodorum* were analyzed (Fig. 2F, G). In the roots and rhizosphere soil of GA, the dominant bacterial genera were *Delftia* (16%) and *Bradyrhizobium* (5%). The dominant bacteria in the roots and rhizosphere soil of GD_understory were *Delftia* (11%) and *Gaiella* (7.3%), respectively. The more abundant bacterial genera in the roots and rhizosphere soil of GD_roadside were *Mycobacterium* (15%) and *Gaiella* (6.5%), respectively. In the root, the proportion of *Mycobacterium* in GD_roadside was higher than that in GD_understory, on the contrary, the proportion of *Delftia* and *Bordetella* in GD_roadside was lower than that in GD_understory. The dominant genera in the roots and rhizosphere soil of GE were *Bordetella* (10%) and *Gaiella* (7%), respectively. These results indicated that there were differences in the composition of bacteria in the roots and rhizosphere soil of different *Geodorum*.

### Fungal community composition of three species of *Geodorum* growing in understory and roadside areas.

**Fig. 3:**
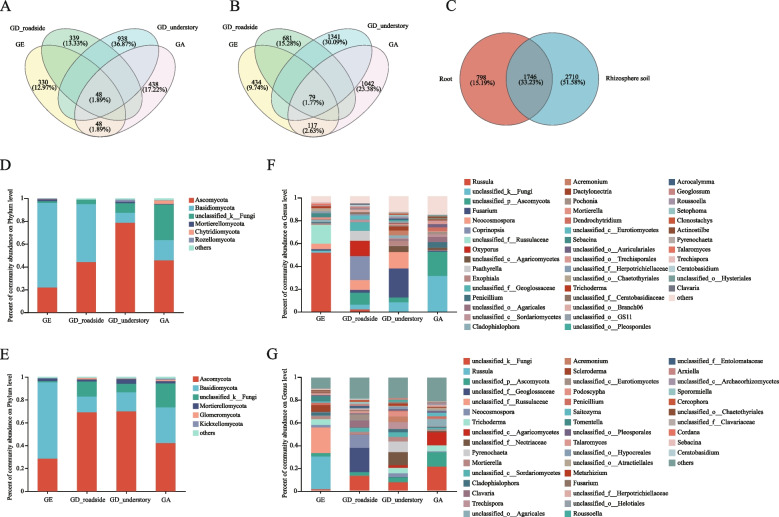
Fungal composition of different *Geodorum* species. **A**, **B** and **C**: Venn diagram of different groups at the OTU level (A: in the roots of GE, GD_roadside, GD_understory and GA groups; B: in the rhizosphere soil of GE, GD_roadside, GD_understory and GA groups; C: in the root and rhizosphere soil groups in all the *Geodorum* species). **D** and **E**: Bar graphs of the bacterial community composition at the phylum level (D: in the roots of *Geodorum*; E: in the rhizosphere soil of *Geodorum*). **F** and **G**: Bar graphs of the bacterial community composition at the genus level (F: in roots of *Geodorum*; **G**: in rhizosphere soil of *Geodorum*). GA, *Geoderma attenuatum*; GD, *Geodorum densiflorum*; GE, *Geodorum eulophioides*; OTU, operational taxonomic unit. In the Venn diagram, the circle for each group represents a taxon, and the area of the circle represents the relative abundance of that taxon in the corresponding group. Overlapping regions represent common taxa between different groups, while non-overlapping regions represent unique taxa

For fungal composition analysis in the roots and rhizosphere soil of *Geodorum*, ITS sequencing was performed on 24 samples, similar to the 16S rRNA approach. A total of 1,169,179 sequences numbers were obtained, with an average length of 253 bp sequences per sample (Table S5). In addition, the highest number of ITS OTUs in the GD_understory was found in both the roots and rhizosphere soil (Table 3). Similarly, there were more fungal species in the rhizosphere soil than in the roots of *Geodorum*. A total of 48 shared ITS OTUs were found in the roots of GE, GD_roadside, GD_understory and GA, and 79 shared ITS OTUs were found in the rhizosphere soil, which was much lower than their shared 16S OTUs (Fig. 3A, B). Overall, only 1,746 (33.23%) OTUs were shared between the roots and rhizosphere soil, which was lower than that of the bacteria (2783: 71.86%), which indicated that there were greater differences between the roots and rhizosphere soil when the fungi were analyzed (Fig. 3C).

**Table 3:** The information on ITS OTU classification statistics in *Geodorum*

Sample	Domain	Kingdom	Phylum	Class	Order	Family	Genus	Species	OTU
GE_S	1	1	10 ± 1abc	30 ± 1ab	66 ± 4abc	126 ± 8ab	195 ± 22ab	258 ± 34ab	499 ± 50bc
GE_R	1	1	9 ± 1bcde	23 ± 1b	48 ± 6bc	92 ± 13b	124 ± 19b	157 ± 29b	261 ± 70c
GD_roadside_S	1	1	10 ± 1ab	28 ± 3ab	64 ± 4abc	139 ± 18ab	213 ± 50ab	275 ± 70ab	560 ± 148bc
GD_roadside_R	1	1	7 ± 1e	22 ± 4b	45 ± 12c	81 ± 26b	109 ± 42b	129 ± 54b	236 ± 112bc
GD_understory_S	1	1	11 ± 1a	36 ± 5a	78 ± 10a	161 ± 21a	284 ± 44a	398 ± 62a	1068 ± 124a
GD_understory_R	1	1	8 ± 2cde	26 ± 7b	57 ± 19abc	113 ± 44ab	177 ± 84ab	231 ± 113b	542 ± 249bc
GA_S	1	1	10 ± 1abcd	31 ± 4ab	70 ± 11ab	139 ± 30ab	223 ± 58ab	284 ± 80ab	790 ± 258ab
GA_R	1	1	8 ± 0de	26 ± 4b	50 ± 6bc	92 ± 13b	121 ± 23b	150 ± 36b	315 ± 63c

*ITS* internal transcribed sequence, *OTU* operational taxonomic unit

A bar plot analysis of the fungal community showed the dominant fungi and their proportions in the roots and rhizosphere soil of *Geodorum*. At the phylum level, Ascomycota, and Basidiomycota had high proportions in the fungal community in both the roots and rhizosphere soil. In addition, the dominant fungal phyla in the roots and rhizosphere soil were basically the same. The proportion of Basidiomycota in the root and rhizosphere soil of GE was 74% and 67%, respectively (Fig. 3D, E). At the genus level, the dominant fungi in the roots of GA were *Penicillium* (4.5%), and in the rhizosphere soil, the dominant fungi was *Trichoderma* (5.3%) (Fig. 3F, G). *Fusarium* accounted for 25% of the roots of GD_understory. It was worth noting that the proportion of *Fusarium* in the root of GD_understory is higher than that in GD_roadside. *Pyrenochaete* (9.4%) was the dominant fungus in the rhizosphere soil of GD_understory. A high proportion of fungi included *Coprinopsis* (21%), *Oxyporus* (16%) and *Neocosmospora* (8.6%) in the roots of GD_roadside. *Russula* (51%) was the dominant fungi in the roots of GE (Fig. 3F). In the rhizosphere soil of GE, *Russula* was also present in the highest abundance (29%) (Fig. 3G).

Twenty-six genus-level mycorrhizal fungi, previously identified in orchids [34], were found in the three *Geodorum* species (Table S6). In all the root samples, the first three genera of related mycorrhizal fungi identified were *Fusarium*, *Russula*, and *Penicillium*. In addition, the proportion of mycorrhizal fungi in the roots of *Geodorum* showed that single mycorrhizal fungi (*Russula* and *Fusarium*) in GE and GD_understory accounted for more than 50%, while no such situation existed in GD_roadside and GA (Fig. S3).

### α-Diversity analysis: Index group difference test

**Fig. 4:**
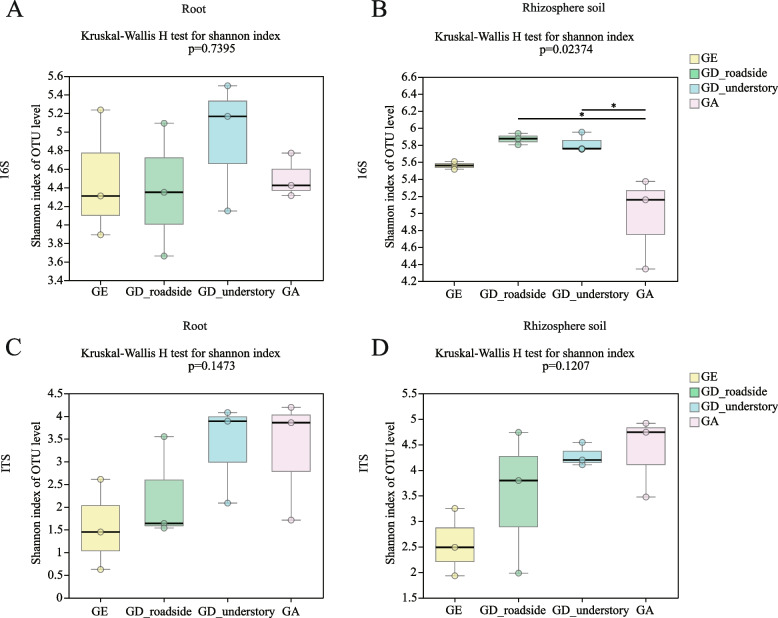
Box plots of the Shannon indices of bacteria and fungi in the root and rhizosphere soil for GE, GD_roadside, GD_understory and GA. A: in the roots of *Geodorum* at the 16S OTU level; B: in the rhizosphere soil of *Geodorum* at the 16S OTU level; C: in the roots of *Geodorum* at the ITS OTU level; D: in the rhizosphere soil of *Geodorum* at the ITS OTU level. *P ≤ 0.05. GA, *Geoderma attenuatum*; GD, *Geodorum densiflorum*; GE, *Geodorum eulophioides*; ITS, internal transcribed spacer; OTU, operational taxonomic unit

Based on the Shannon index, GD_understory had the highest level of 16S OTUs among the roots of *Geodorum*, but there was no significant difference compared with the other *Geodorum* (Fig. 4A). In contrast, the GD that grew in the understory and roadside had significantly higher levels of 16S OTUs than those in the rhizosphere soil of GA among the three species of *Geodorum* (Fig. 4B). It was also notable that GE had the lowest diversity in its ITS OTU Shannon diversity analysis. This was found in both its roots and rhizosphere soil (Fig. 4C, D). Low fungal diversity in GE and a high diversity of ITS OTUs were observed in GD_understory and GA.

### NMDS and ANOSIM analysis

**Fig. 5:**
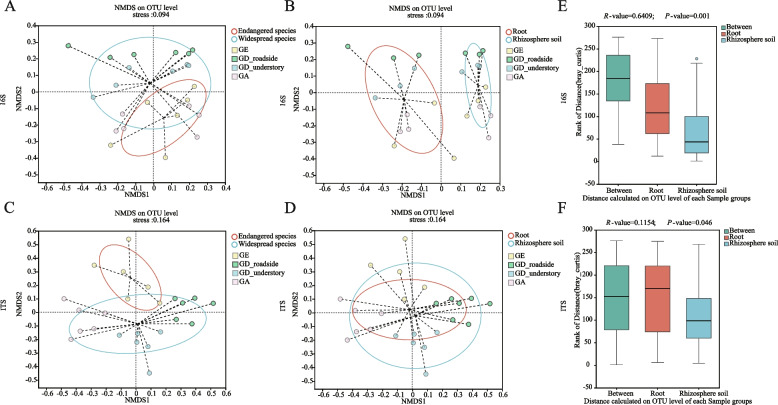
NMDS and ANOSIM analysis. **A**, **B**, **C** and **D**: NMS analysis (A: in the endangered and widespread species groups at the 16S OTU level; B: in the root and rhizosphere soil groups at the 16S OTU level; C: in the endangered and widespread species groups at the ITS OTU level; D: in the root and rhizosphere soil groups at the ITS OTU level). E and F: ANOSIM analysis between the root and rhizosphere soil groups (E: at the 16S OTU level; F: at the ITS OTU level). ANOSIM, analysis of similarity; ITS, internal transcribed spacer; NMDS, non-metric multidimensional scaling; OTU, operational taxonomic unit

β-diversity was analyzed using non-metric multidimensional scaling (NMDS) to compare bacterial and fungal differences between roots, rhizosphere soil, and between endangered (GE) and widespread (GD and GA) species. At the 16S OTU level, there was no significant separation in the bacteria between the endangered species and widespread species groups, while there was a significant separation between the root and rhizosphere soil groups (Fig. 5A, B). This indicates a greater environmental than species influence on bacterial composition. In fungi, there was a significant difference between those groups of species that were endangered compared with those that were widespread (Fig. 5C, D). Analysis of similarity (ANOSIM) results revealed greater differences between root and rhizosphere soil than among *Geodorum* species at the bacterial 16S OTU level (*R*-value = 0.6409; *P*-value = 0.001) (Fig. 5E). As for fungi, the differences in different species of *Geodorum* were greater than the difference between the root and rhizosphere soil groups (*R*-value = 0.1154; *P*-value = 0.046) (Fig. 5F), which was consistent with the results of NMDS analysis.

### The differences in bacterial and fungal composition between the roots and rhizosphere soil and between endangered and widespread species of *Geodorum*

**Fig. 6:**
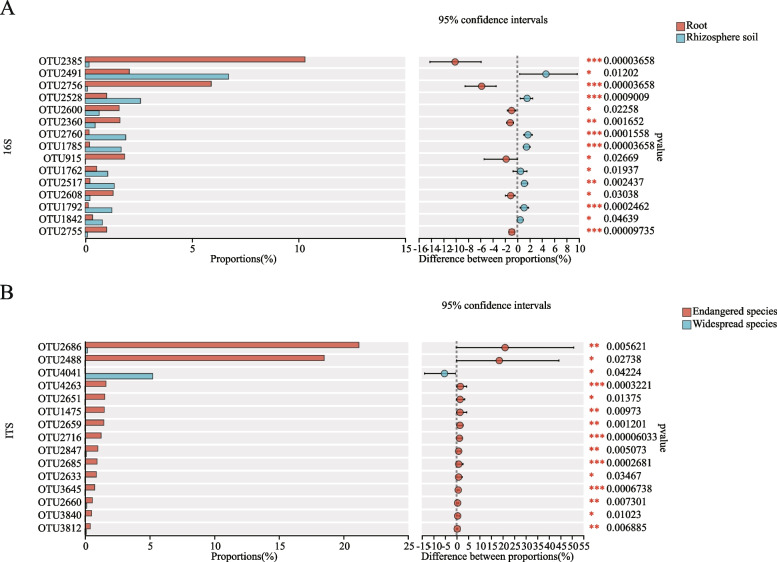
Bar graph of the species difference test. A: between the root and rhizosphere soil groups at the 16S OTU level; B: between the endangered and widespread species groups at the ITS OTU level. The positive and negative difference in mean relative abundance represents the abundance of OTUs in the corresponding group. *P ≤ 0.05. **0.01 ≤ *P* ≤ 0.05. ***0.001 ≤ *P* ≤ 0.01. ITS, internal transcribed spacer; OTU, operational taxonomic unit

A significance test between groups identified microorganisms with significantly different abundances, aiding further analysis of the microbial composition of *Geodorum*. At the level of 16S OTU classification, 15 OTUs with significant differences were obtained by a Wilcoxon rank sum test. OTU2385 (g__Delftia), OTU2756 (g__Bordetella), OTU2600 (g__Bradyrhizobium), OTU2360 (g__Pseudorhodoplanes), OTU915 (g__Burkholderia-caballeronia-paraburkholderia), OTU2608 (g__norank_f__Hyphomicrobiaceae), and OTU2755 (g__Pseudomonas) were significantly more abundant in the roots than in the rhizosphere soil, and the proportion of OTU2491 (g__norank_f__Xanthobacteraceae), OTU2529 (g__norank_f__norank_o__norank_c__subgroup_22), OTU2760 (g__Gaiella), OTU1785 (g__Bacillus), OTU1762 (g__MND1), OTU2517(g__norank_f__67-14), OTU1792 (g__Gaiella) and OTU1842 (g__norank_f__norank_o__Gaiellales) in the rhizosphere soil was significantly higher than that in the roots (Fig. 6A). In fungi, only OTU4041 (g__unclassified_f__Geoglossaceae) was higher in the widespread than in the endangered species, and the other 14 OTUs were significantly higher in the endangered than in the widespread species, particularly OTU2686 (g__Russula) and OTU2488 (g__unclassified_f__Russulaceae) (Fig. 6B).

### LEfSe analysis

**Fig. 7:**
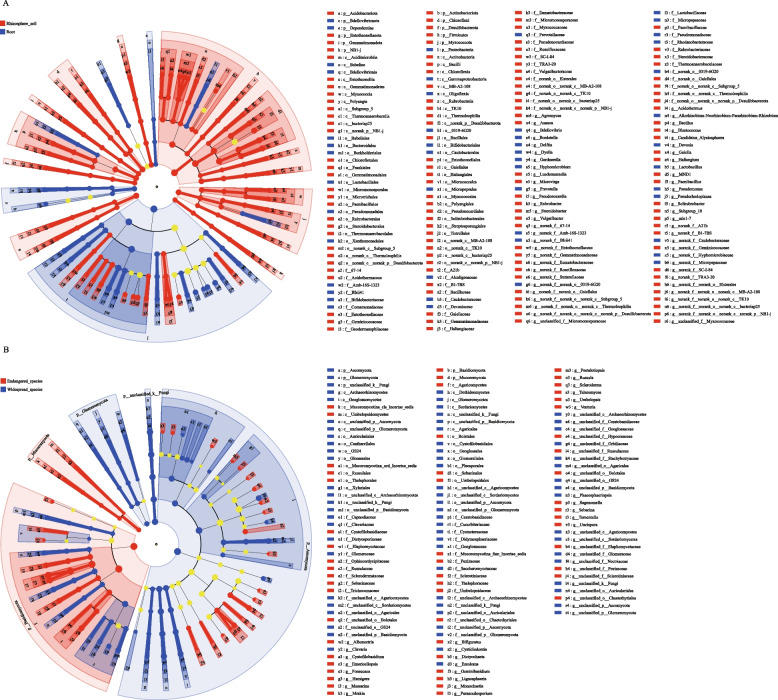
A: LEfSe analysis of bacterial composition in GA, GE,GD_understory and GD_roadside on the phylum to genus level. B: LEfSe analysis of fungal composition in GA, GE, GD_understory and GD_roadside on the phylum to genus level. ITS, internal transcribed spacer; OTU, operational taxonomic unit

LEfSe analysis was used to identify the biomarker species in different *Geodorum*. In terms of bacterial composition, GE had four and GA had three dominant bacterial genera, whereas GD_roadside and GD_understory exhibited 13 and 14, respectively (Fig. 7A). Of the fungal compositions examined, GD_understory exhibited the greatest prevalence of fungi, with a count of 22. Conversely, GE had 10, GD_roadside had 9, and GA had 11 (Fig. 7B). At the bacterial phylum level, Acidobacteriota was enriched on GE, while Bacteroidota and Nitrospirota were enriched on GD_understory. At the level of the fungal phylum, the GE concentration of microorganisms comprises Basidiomycota and Mucoromycota. Chytridiomycota and Kickxellomycota exhibited GA enrichment, while Glomeromycota was enriched in GD_roadside enrichment and Ascomycota was enriched in GD_understory.

### Co-occurrence network analysis

The co-occurrence network map reflects the coexistence of species in different samples. By analyzing the species abundance information among different samples, the co-existence patterns of microbial species in different samples can be observed and understood (Figure S4). Twenty-two 16S OTUs were detected within the roots of at least two species of *Geodorum*, while 17 were found in the rhizosphere soil of at least two species of *Geodorum* (Figure S4A, B). Moreover, in the roots and rhizosphere soil, OTU2687 (g__Mycobacterium), OTU2666 (g__Bradyrhizobium), OTU2491 (g__norank_f__Xanthobacteraceae) and OTU2528 (g__Solirubrobacter) were their co-occurring 16S OTUs. Seven 16S OTUs detected in the rhizosphere soil of *Geodorum* were not found to be associated with its roots, whereas fourteen 16S OTUs detected in the roots were not associated with the rhizosphere soil (Figure S4C). Eight ITS OTUs were detected within the roots of at least two species of *Geodorum*, while nine were found in the rhizosphere soil of at least two species of *Geodorum* (Figure S4D, E). 19 of the ITS OTUs of the endangered species were not associated with the widespread species, and 38 of the widespread species were not associated with the endangered species, which also reflected the low number of highly abundant fungi in the endangered species (Figure S4F). Eight 16S OTUs were associated in the rhizosphere soil and roots of GA and eight in the rhizosphere soil and roots of GD_understory. The lowest number was 5 in GD rhizosphere soil and roots, while the highest number was 11 in the rhizosphere soil and roots of GE (Figure S5). Only two ITS OTUs were associated in the rhizosphere soil and roots of GA, whereas 12 ITS OTUs were present in the rhizosphere soil and roots of GE (Figure S6).

## Discussion

The growth and development of these plants are closely linked to soil microorganisms, and analyzing the composition of these microorganisms plays a crucial role in promoting plant conservation [2]. This study analyzed the composition of bacteria and fungi in the roots and rhizosphere soil of GE, GD_roadside, GD_understory, and GA. To explore the microbial diversity of roots and rhizosphere soil of *Geodorum* by detecting microbial species in roots and rhizosphere soil in different habitats, and to provide reference for future conservation and breeding research of *Geodorum*.

### Differences in the microbial composition of roots and rhizosphere soil in the roadside and understory habitats

In the 16S and ITS OTU taxonomy, the number of microorganisms in each sample at each taxonomic level was analyzed (Table 2 and 3). Although there was no clear difference in the number of 16S OTUs in the rhizosphere soil of GD_roadside and GD_understory, there were more 16S OTUs in the roots of GD_understory than in the GD_roadside. At the phylum level, Proteobacteria, Actinobacteriota, Acidobaceriota, Myxococcota were the most abundant bacteria in the roots and rhizosphere soil of *Geodorum* (Fig. 2D, E). In the LEfSe analysis, Acidobacteriota was enriched on GE, while Bacteroidota and Nitrospirota were enriched on GD_understory. The soil in GE was more acidic, which may have contributed to the higher abundance of Acidobacteriota in roots and rhizosphere soil of GE than that in GD_understory, GD_roadside and GA. Some studies have found that soil with low pH was more conducive to the growth and abundance of Acidobacteriota, while soil with high pH has a negative effect on Acidobacteriota [35]. The high abundance of Bacteroidota and Nitrospirota might be attributed to the high organic matter and nitrogen content in the soil of GD_understory [36].

In addition, the GD_understory had more ITS OTUs than the GD_roadside in both the roots and rhizosphere soil. This indicated that the effect of understory habitat on soil microbial structure is primarily on fungi, which was consistent with the structure of research on the influence of understory vegetation on the soil microbial community structure [37]. Soils rich in organic matter typically facilitate increased microbial abundance [38]. The soil organic matter content in GD_understory was significantly higher than that in GD_roadside, and the soil in GD_roadside was relatively poor, resulting in lower microbial abundance than that in GD_understory (Table S1).

GE, GD_roadside, GD_understory and GA had similar dominant bacterial and fungal phyla in the roots and rhizosphere soil. Proteobacteria, Actinobacteriota and Acidobacteriota were the dominant bacterial phyla of the roots and rhizosphere soil in *Geodorum*. These bacterial phyla promote the decomposition of organic material, aiding the plants in absorbing elements like nitrogen, phosphorus (P), and potassium (K) [39–42]. At the genus level, the dominant bacteria in the rhizosphere soil and roots were different. *Delftia* and *Bordetella* were the dominant genera in the roots of *Geodorum*, while *Gaiella* and *Solirubrobacter* were the dominant genera in the rhizosphere soil. In the co-occurrence analysis, 22 16S OTUs and 17 16S OTUs were identified in the roots and rhizosphere soil of at least two species *Geodorum*, respectively. It may reflect a wide range of adaptations of some microorganisms to environmental conditions, or it may be caused by common characteristics between samples, suggesting that these microorganisms may have a wide range of adaptive adaptations, with similar patterns of presence among *Geodorum*. Similarly, Ascomycota and Basidiomycota were the dominant fungal phyla in the roots and rhizosphere soils of GD_roadside, GD_understory, and GE. Additionally, orchid mycorrhizal fungi are derived from the root endophytic fungi, and many Ascomycota and Basidiomycota fungi are endophytic [43–45]. The growth of orchids depends on the assistance of mycorrhizal fungi in the absorption of various elements in the soil, and mycorrhizal fungi can promote the germination of orchid seeds [46, 47]. Compared with GD_roadside and GA, the roots of GD_understory and GE had relatively single mycorrhizal fungi (Table S6), which might be related to the specific selection of the fungi on the host [48], but their specificity needs to be further studied.

In the root, the proportion of *Mycobacterium* in GD_roadside was higher than that in GD_understory, on the contrary, the proportion of *Delftia* and *Bordetella* in GD_roadside was lower than that in GD_understory. *Delftia* can promote plant growth and produce siderophores, which enables it to serve as an endophyte that facilitates the absorption of iron by *Geodorum * [49, 50]. The root of GD_roadside had a higher proportion of *Mycobacterium* than the root of GD_understory, but the reason for this difference has not been determined. The dominant fungi in rhizosphere soil and roots of GD_understory and GD_roadside differed considerably at the genus level. In the roots of GD_understory and GD_roadside, their primary types of endophytic fungi also differed at the genus level with the endophytic fungi of GD_roadside primarily related to *Coprinopsis*, *Oxyporus*, and *Neocosmospora*. Nevertheless, the endophytic fungi in GD_understory were primarily identified as *Fusarium* and *Neocosmospora*. In recent studies, *Fusarium* have been found to cause root rot and stem rot in plant, resulting in poor plant growth, yellow and wilting leaves, and even death in severe cases [51]. However, *Fusarium* has been identified as a mycorrhizal fungus in some Orchidaceae species, including *Dendrobium officinale* and *Paphiopedilum * [52, 53]. *Fusarium* can improve the absorption of P, K and calcium and the activity of various antioxidant enzymes, which can enhance the adaptability of orchids to manage the external environment [54]. Since orchids often grow in environments with high humidity and temperature, these conditions are often also suitable for the propagation and infection of *Fusarium* in plants [55]. The high nitrogen content in the soil of GD_understory may also be one of the reasons for the growth and reproduction of *Fusarium * [56]. *Oxyporus* enhances the decomposition of organic matter, thereby improving nutrient conversion efficiency It can also repair soil to some degree and reduce the damage to plants from heavy metals in soil [57, 58]. As a orchid plant, GD_understory may have a special nutritional relationship with *Fusarium*, and its root may form mycorrhizal with *Fusarium* and rely on this fungus to provide nutrition, but this needs further study. In addition, *Neocosmospora*, a plant endophyte, is associated with mold in plants, thus, affecting plant growth [59]. Furthermore, α-diversity analysis reveals that both bacterial and fungal diversity in the roots of GD_understory surpass those in GD_roadside, as indicated by 16S and ITS OTU counts.

### Differences in fungal composition in the roots and rhizosphere soil between endangered and widespread species

The characteristics of fungi in endangered and widespread species were analyzed to understand whether the process of GE becoming endangered was related to the fungal composition in the roots and rhizosphere soil. Analysis revealed that GE exhibited the lowest number of ITS OTUs, potentially impeding its growth. The fungal community in the soil is closely related to the growth of plants, and the diversity of soil fungi can improve the resistance of plants to the environment, which can not only promote the growth of plants but also maintain the stability of ecosystems [60]. Many mycorrhizal fungi of the Orchidaceae are members of Ascomycota, Basidiomycota and Mortierellomycota, and according to the mycorrhizal fungi identified in orchids, fewer ITS sequences associated with Ascomycota were screened in the roots of GE than in the roots of GA and GD, including *Aspergillus*, *Penicillium* and *Fusarium*. The diversity, abundance and composition of soil microorganisms affect the stability of soil ecosystems, the efficiency of soil nutrient cycling, and the healthy growth of plants [61]. This suggested that lower fungal diversity may be one of the reasons why GE is an endangered species. Although Basidiomycota is the most common mycorrhizal fungal phylum in orchids, the high abundance of Basidiomycota in the roots or rhizosphere soil of GE is owing to the presence of a single high abundance of *Russula * [62, 63]. This suggested that the fungi of GE are primarily associated with *Russula*, aligning with the differences observed between widespread and endangered species. *Russula* typically thrives in soils that are abundant in organic matter, such as forest or woodland soils. It can also grow in soils that are either acidic or neutral, although it is comparatively less well-suited to alkaline soils [64]. Therefore, acidic soils in GE habitats may contribute to the high abundance of *Russula*. In addition to soil pH, since GE grows in the understory, the composition of microorganisms is also affected by the upper trees, and the dominance of *Russula* is also affected by the composition of the tree community, especially the association of its ectomycorrhizae with tree roots. Some tree species may have a preference for a symbiotic relationship with *Russula*, resulting in its dominance in the mycorrhizal community [65]. Other previous research indicates a negative correlation between *Russula* abundance and overall fungal diversity because mycorrhizal metabolites and proteins had selective antimicrobial (anti-microbial) effects, especially against rhizosphere bacterial species, leading to a decreasing trend in the diversity of fungal and bacterial species in the mycorrhizal sphere, possibly contributing to the low fungal diversity observed in GE's root and rhizosphere soil [66]. Although heterotrophic effects that depend on *Russula* are suspected on violet bird's-nest (*Limodorum abortivum*), *L. trabutianum* and *L. brulloi * [67, 68], there is also evidence that a single mycorrhizal fungus can have a large limiting effect on plant growth and distribution [69]. Interestingly, the exclusivity of a single fungus has been reported in some orchids or other plants, particularly in mycoheterotrophic angiosperms, which could indicate that *Russula* is associated with low fungal diversity in the roots of GE [70–72]. Molecular identification studies have shown that GE has a strong specificity for fungi, and this bias is also present in other Orchidaceae plants, which may be due to the specific selection of fungi on their hosts or orchids on fungi [73, 74]. Thus, the high abundance of *Russula* in GE may be its specific selection for fungi. The types of colonization by mycorrhizal fungi on orchids primarily include specific colonization, extensive colonization, and specific-extensive facultative colonization. There were differences in the composition of fungal species between GD_understory and GD_roadside, particularly those that had been identified as mycorrhizal fungi in other orchids, which could indicate that there were extensive colonization types of GD and fungi.

The g_unclassified_f__Geoglossaceae abundance of GE was significantly lower than those of widespread species. Currently, it has been found that Geoglossomycetes can form hyphal coils in the root cortex of plants, which could promote the exchange of nutrients between Geoglossomycetes and their roots [75]. The abundance of *Fusarium* and *Neocosmospora*, which could support the growth, nutrient absorption and ecological adaptability of Orchidaceae, were significantly lower in the root of GE than in the root of GD and GA [76]. As a result, GE has a low abundance of fungi in its roots compared with the widespread species, which could affect its ability to reproduce. It is still controversial whether there is a relationship between the rarity of orchids and the specificity of fungi. Currently, it has been reported that the endangered grand spider orchid (*Caladenia huegelii*) is threatened because of its high specificity with mycorrhizal fungi, which affects its mycorrhizal establishment with other fungi [77]. Studies have also shown that GE has small seeds and a low germination rate, which prevents the population of GE from expanding [3]. The tiny seeds and lack of endosperm in orchids, such as pigeon orchids (*Dendrobium crumenatum*), contribute to their low fecundity, which is one of the reasons why they are endangered [78]. The endangered status of GE may be attributed to the specific symbiosis between GE and *Russula*, coupled with the absence of other beneficial fungi, leading to reduced growth and reproductive capabilities. Consequently, orchids require endophytic fungi to promote seed germination under natural conditions, and GE could lack the assistance of endophytic fungi to promote its growth owing to its low fungal diversity, which could further contribute to its endangered state.

### Factors that affect low microbial diversity

The α-diversity analysis of bacteria and fungi in the root and rhizosphere soil of *Geodorum* indicated that GA had the lowest bacterial diversity in the rhizosphere soil (Figure S2). In low-altitude areas, soil carbon, N, and P contents generally rise with increasing altitude, peaking in mid-altitude regions before decreasing in higher-altitude areas. Similarly, bacterial diversity tends to increase up to a certain altitude and then decreases at higher altitudes [79–81]. The variation in climate and precipitation between Baise City and Chongzuo City suggests that altitude is a key factor influencing soil microbial diversity in GA, warranting further investigation. In line with the above findings, α-diversity analysis revealed the lowest ITS OTU levels in GE's roots and rhizosphere soil, suggesting minimal fungal diversity, possibly due to the dominance of the fungus *Russula*. The results of an NMDS analysis showed that the difference in the levels of 16S OTUs between the roots and rhizosphere soil was greater than that between the endangered and widespread species. In contrast, there was a greater difference in the levels of ITS OTUs between the endangered and widespread species than between the roots and rhizosphere soil. This indicates that there is some degree of independence of bacterial characteristics between the roots and rhizosphere soil and reflects that the fungal composition of soil may relate to the fungal species in the roots of *Geodorum*. According to the results of intergroup differences and co-occurrence analysis, the proportions of OTU2385 (g_Delftia), OTU2756 (g_Bordetella) and OTU2755 (g_Pseudomonas) in the roots were significantly higher than those in the rhizosphere soil (*P* ≤ 0.001), which may be related to soil properties, microbial relationships, microbial physiological adaptations, plant regulation, etc. [82].

## Conclusions

In this study, the bacterial and fungal diversity of the roots and rhizosphere soil in GE, GD_roadside, GD_understory and GA were analyzed. The study revealed that GA's rhizosphere soil, located at the lowest altitude compared to GD and GE, exhibited the lowest 16S OTU levels. A low-altitude habitat could be one of the reasons for the low bacterial diversity in the rhizosphere soil of GA. The lower fungal and bacterial diversity in the root of GD_roadside compared to GD_understory may be related to the lower soil organic matter and nitrogen content in GD_roadside, which may hinder the growth and reproduction of *Geodorum*. Additionally, α-diversity analysis indicated the lowest ITS OTU levels in both the roots and rhizosphere soil of GE. In GE, the dominance of the fungus *Russula* in both roots and rhizosphere soil correlates with low fungal diversity, potentially contributing to its endangered status. GE growth in acidic soils and understory environments may be related to the high abundance of *Russula* in roots and rhizosphere soils. In contrast, there was a variety of dominant fungi in the roots and rhizosphere soil of widespread species, including *Fusarium* and *Neocosmospora*, *Nectriaceae*, *Coprinopsis*, and *Oxyporus*, among others, which may have some influence on the distribution and reproduction of *Geodorum*.

### Supplementary Information

**Supplementary Material 1.**

**Supplementary Material 2.**

**Supplementary Material 3.**

**Supplementary Material 4.**

**Supplementary Material 5.**

**Supplementary Material 6.**

**Supplementary Material 7:**

**Supplementary Material 8:**

**Supplementary Material 9:**

**Supplementary Material 10:**

**Supplementary Material 11:**

**Supplementary Material 12:**

## Data Availability

The sequencing data have been deposited in the NCBI with Sequence Read Archive (SRA) accession No. PRJNA953938.

## References

[1] Xu A, Chai S, Wei X (2022). Advances in Studies of Geodorum G. Jacks. (Orchidaceae). Journal of Guangxi Academy of Sciences.

[2] Saleem, M., J. Hu, and A. Jousset, More Than the Sum of Its Parts: Microbiome Biodiversity as a Driver of Plant Growth and Soil Health, in Annual Review Of Ecology, Evolution, And Systematics, D.J. Futuyma, Editor. 2019. p. 145,163–168.

[3] Wei H (2018). Analysis of resource status and endangered causes of the extremely endangered plant Geodorumeulophioides in Guizhou Province. Journal of Mountain Agriculture and Biology.

[4] Lin, M., et al., The Effect of Plant Geographical Location and Developmental Stage on Root-Associated Microbiomes ofGymnadenia conopsea. Frontiers In Microbiology, 2020. 11. 10.3389/fmicb.2020.01257 PMC7314937 32625183

[5] Böhmer M (2020). Identification of bacterial and fungal communities in the roots of orchids and surrounding soil in heavy metal contaminated area of mining heaps. Appl Sci (Basel).

[6] Yang Y, Liu Z (2005). The role and application of mycorrhizal fungi in the growth and development of Orchidaceae. Seed.

[7] Favre-Godal Q (2020). Orchids and their mycorrhizal fungi: an insufficiently explored relationship. Mycorrhiza.

[8] Cozzolino S (2003). Variation at a chloroplast minisatellite locus reveals the signature of habitat fragmentation and genetic bottlenecks in the rare orchid Anacamptis palustris (Orchidaceae). Am J Bot.

[9] Kang JY (2015). Dendrobium SSR Markers play a good role in genetic diversity and phylogenetic analysis of Orchidaceae species. Sci Hortic.

[10] Zhang G-Q (2017). The Apostasia genome and the evolution of orchids. Nature.

[11] Ping Z (2012). Main characters and techniques on reproduction and cultivation of Dendrobium candidum in Guangdong area. Guangdong Agricultural Sciences.

[12] Taylor DL (2003). Divergence in mycorrhizal specialization within Hexalectris spicata (Orchidaceae), a nonphotosynthetic desert orchid. Am J Bot.

[13] Mujica MI (2021). Nutrients and fungal identity affect the outcome of symbiotic germination in Bipinnula fimbriata (Orchidaceae). Symbiosis.

[14] Qin J (2021). Mycorrhizal fungal partners remain constant during a root lifecycle of Pleione bulbocodioides (Orchidaceae). Journal of Fungi.

[15] Ling T (2022). Study on rhizosphere microorganism diversity of a myco-heterotrophic orchid endemic to North China, Holopogon pekinensis XY Mu & Bing Liu. Plant Science Journal.

[16] Xiong W (2017). Distinct roles for soil fungal and bacterial communities associated with the suppression of vanilla Fusarium wilt disease. Soil Biol Biochem.

[17] Carbajal-Valenzuela IA (2022). Microbial Diversity in Cultivated and Feral Vanilla Vanilla planifolia Orchids Affected by Stem and Rot Disease. Microb Ecol.

[18] Liang J (2022). Structure and diversity of mycorrhizal fungi communities of different part of Bulbophyllum tianguii in three terrestrial environments. Front Plant Sci.

[19] Zhu M (2022). Effect of cultivation mode on bacterial and fungal communities of Dendrobium catenatum. BMC Microbiol.

[20] Lin, W., et al., Comparative Reproductive Biology of a Rare Endemic Orchid and its Sympatric Congeners in Southwestern China. 2012, Florida International University.

[21] ZhangWufan, A Primary of Mycorrhizal Fungi and Seed Germination of Geodorum G.Jacks.(Orchidaceae). 2014, Beijing Forestry University.

[22] Yan-yan P (2020). Cytology Studies on Embryo Sac and Embryo Development of Geodorum recurvum. Journal of Tropical and Subtropical Botany.

[23] Lin, W., et al., Comparative Reproductive Biology of a Narrowly Endemic Orchid Geodorum eulophioides, and its Sympatric Congeners in Southwestern China.

[24] Walkley A, Black I.A (1934). an examination of the degtjareff method for determining soil organic matter, and a proposed modification of the chromic acid titration method. Soil Science.

[25] Holford ICR (1997). Soil phosphorus: its measurement, and its uptake by plants. Soil Research.

[26] Gardes M, Bruns TD (1993). ITS primers with enhanced specificity for basidiomycetes-application to the identification of mycorrhizae and rusts. Mol Ecol.

[27] Chen S (2018). fastp: an ultra-fast all-in-one FASTQ preprocessor. Bioinformatics.

[28] Magoč T, Salzberg SL (2011). FLASH: fast length adjustment of short reads to improve genome assemblies. Bioinformatics.

[29] Edgar RC (2013). UPARSE: highly accurate OTU sequences from microbial amplicon reads. Nat Methods.

[30] Wang Q (2007). Naive Bayesian classifier for rapid assignment of rRNA sequences into the new bacterial taxonomy. Appl Environ Microbiol.

[31] Conway JR (2017). UpSetR: an R package for the visualization of intersecting sets and their properties. Bioinformatics.

[32] Baczkowski A, Joanes D, Shamia G (1998). Range of validity of α and β for a generalized diversity index H (α, β) due to Good. Math Biosci.

[33] Clarke KR (2006). Non-parametric multivariate analyses of changes in community structure. Aust J Ecol.

[34] Wang M (2021). New insights into orchid mycorrhizal fungi research. Guihaia.

[35] Navarrete AA (2013). Acidobacterial community responses to agricultural management of soybean in Amazon forest soils. FEMS Microbiol Ecol.

[36] Rousk J, Brookes PC, Bååth E (2009). Contrasting Soil pH Effects on Fungal and Bacterial Growth Suggest Functional Redundancy in Carbon Mineralization. Appl Environ Microbiol.

[37] Chen W (2023). The removal of understory vegetation can rapidly alter the soil microbial community structure without altering the community assembly in a primary tropical forest. Geoderma.

[38] Cui, J., et al., Investigating the effects of organic amendments on soil microbial composition and its linkage to soil organic carbon: A global meta-analysis. Science of The Total Environment, 2023. 894. 10.1016/j.scitotenv.2023.164899 37343853

[39] Starke R (2016). Bacteria dominate the short-term assimilation of plant-derived N in soil. Soil Biol Biochem.

[40] Choi K, Khan R, Lee S-W (2021). Dissection of plant microbiota and plant-microbiome interactions. J Microbiol.

[41] Xu R (2022). Responses of endophytic bacterial communities in rice roots to phosphorus deficiency at the seedling stages. Eur J Soil Biol.

[42] Arsyadi A (2023). A Nitrate-Transforming Bacterial Community Dominates in the Miscanthus Rhizosphere on Nitrogen-Deficient Volcanic Deposits of Miyake-jima. Microorganisms.

[43] Dearnaley JD, Martos F, Selosse M-A (2012). Orchid mycorrhizas: molecular ecology, physiology, evolution and conservation aspects. Fungal associations.

[44] Selosse M-A (2022). The Waiting Room Hypothesis revisited by orchids: were orchid mycorrhizal fungi recruited among root endophytes?. Ann Bot.

[45] Xu, Z.-X., et al., Symbiosis between Dendrobium catenatum protocorms and Serendipita indica involves the plant hypoxia response pathway. Plant Physiology, 2023: p. kiad198. 10.1093/plphys/kiad198 PMC10315314 36988071

[46] Pandey M (2013). A narrowly endemic photosynthetic orchid is non-specific in its mycorrhizal associations. Mol Ecol.

[47] Chen Y, Xing X, Guo S (2017). Nutritional relationships between orchids and mycorrhizal fungi: a review. Mycosystema.

[48] Zhang, W., A primary study of mycorrhizal fungi and seed germination of geodorum G.jacks.(orchidaceae). 2014, Beijing Forestry University.

[49] Morel, M.A., et al., Delftia sp. JD2: a potential Cr (VI)-reducing agent with plant growth-promoting activity. Archives of microbiology, 2011. 193: p. 63–68. 10.1007/s00203-010-0632-2 20857088

[50] Ubalde M.C (2012). The versatility of Delftia sp. isolates as tools for bioremediation and biofertilization technologies. Current microbiology.

[51] Pérez Mora, W.H., et al., Thiamine-induced resistance in carnation against Fusarium oxysporum f. sp dianthi and mode of action studies based on the proteomics analysis of root tissue. Scientia Horticulturae, 2024. 323.

[52] CHEN Baoling, Y.K., G.J. , TANG Qing, SU Lihua, WANG Xiaoyu,, and L. Dingjian, Effects of Wild Paphiopedilum Mycorrhizal Fungi on Growth and Physiological Indexes of Paphiopedilum hirsutissimum Seedlings. Journal of Tropical and Subtropical Botany, 2022. 30(1): p. 88–96.

[53] Jiang J (2019). Fusarium oxysporum KB-3 from Bletilla striata: an orchid mycorrhizal fungus. Mycorrhiza.

[54] Yang, H., Screening and Functional Characterization of Endophytic mycorrhizal fungus in Dendrobium officinale. 2015.

[55] Chaparro JM (2012). Manipulating the soil microbiome to increase soil health and plant fertility. Biol Fertil Soils.

[56] Wen YC (2020). Long-term fertilization alters soil properties and fungal community composition in fluvo-aquic soil of the North China Plain. Sci Rep.

[57] Srivastava PK (2011). Biological removal of arsenic pollution by soil fungi. Sci Total Environ.

[58] Jia T (2021). Litter decomposition of Imperata cylindrica in a copper tailing areas with different restoration history: fungal community dynamics and driving factors. Front Microbiol.

[59] Chen P (2023). Synergistic effect of Bacillus subtilis and Paecilomyces lilacinus in alleviating soil degradation and improving watermelon yield. Front Microbiol.

[60] Liu S (2022). Phylotype diversity within soil fungal functional groups drives ecosystem stability. Nature Ecology & Evolution.

[61] Wagg C (2014). Soil biodiversity and soil community composition determine ecosystem multifunctionality. Proc Natl Acad Sci.

[62] Gao Y (2022). Mycorrhizal fungus Coprinellus disseminatus influences seed germination of the terrestrial orchid Cremastra appendiculata (D. Don) Makino. Scientia Horticulturae.

[63] Li J (2021). Effects of different mycorrhizal fungi on growth and root system of plug seedlings of Dendrobium officinale. Acta Agric Boreali-Occident Sin.

[64] Yu, F., et al., Bacterial Community Selection of Russula griseocarnosa Mycosphere Soil. Frontiers in Microbiology, 2020. 11. 10.3389/fmicb.2020.00347 PMC7109302 32269551

[65] Põlme S (2017). Host preference and network properties in biotrophic plant–fungal associations. New Phytol.

[66] Yu WY (2021). Microbial community associated with ectomycorrhizal Russula symbiosis and dominated nature areas in southern China. FEMS Microbiol Lett.

[67] Girlanda M (2006). Inefficient photosynthesis in the Mediterranean orchid Limodorum abortivum is mirrored by specific association to ectomycorrhizal Russulaceae. Mol Ecol.

[68] Valadares RB (2021). A transcriptomic approach provides insights on the mycorrhizal symbiosis of the mediterranean orchid Limodorum abortivum in nature. Plants.

[69] Leake JR (2004). Myco-heterotroph/epiparasitic plant interactions with ectomycorrhizal and arbuscular mycorrhizal fungi. Curr Opin Plant Biol.

[70] Bidartondo M, Bruns T (2002). Fine-level mycorrhizal specificity in the Monotropoideae (Ericaceae): specificity for fungal species groups. Mol Ecol.

[71] Selosse M.A (2002). Communities and populations of sebacinoid basidiomycetes associated with the achlorophyllous orchid Neottia nidus‐avis (L.) LCM Rich. and neighbouring tree ectomycorrhizae. Molecular Ecology.

[72] Bidartondo M.I (2003). Specialized cheating of the ectomycorrhizal symbiosis by an epiparasitic liverwort. Proceedings of the Royal Society of London. Series B: Biological Sciences.

[73] van Der Heijden MG (2015). Mycorrhizal ecology and evolution: the past, the present, and the future. New Phytol.

[74] Brundrett MC, Tedersoo L (2018). Evolutionary history of mycorrhizal symbioses and global host plant diversity. New Phytol.

[75] Baba T (2021). Genetic variations and in vitro root-colonizing ability for an ericaceous host in Sarcoleotia globosa (Geoglossomycetes). Fungal Biol.

[76] Wu J, Qian J, Zheng S (2002). A preliminary study on ingredient of secretion from fungi of orchid mycorrhiza Ying Yong Sheng tai xue bao. The Journal of Applied Ecology.

[77] Swarts ND (2010). Ecological specialization in mycorrhizal symbiosis leads to rarity in an endangered orchid. Mol Ecol.

[78] Vellupillai M, Swarup S, Jin Goh C (1997). Histological and protein changes during early stages of seed germination in the orchid, Dendrobium crumenatum. Journal of Horticultural science.

[79] Mao, W., Variations in soil bacterial community from soil samples along the altitude gradient at the upper reaches of the Heihe River, Northwestern China. 2013.

[80] Siles JA, Margesin R (2017). Seasonal soil microbial responses are limited to changes in functionality at two Alpine forest sites differing in altitude and vegetation. Sci Rep.

[81] Xiran S (2021). Responses of soil nutrients and microbial community to altitude in typical Pinus yunnanensis forest at rocky desertification region. Acta Agriculturae Zhejiangensis.

[82] Deng Z (2022). Effects of Plant Fine Root Functional Traits and Soil Nutrients on the Diversity of Rhizosphere Microbial Communities in Tropical Cloud Forests in a Dry Season. Forests.

