# Supplementary material for: The structure and diversity of bacteria and fungi in the roots and rhizosphere soil of three different species of Geodorum
Source: BMC Genomics. 2024 Feb 28;25:222. doi: 10.1186/s12864-024-10143-2 (PMC10903027; doi:10.1186/s12864-024-10143-2)
Supplement: Supplementary file 1 — Supplementary Material 1. [file 12864_2024_10143_MOESM1_ESM.pdf]

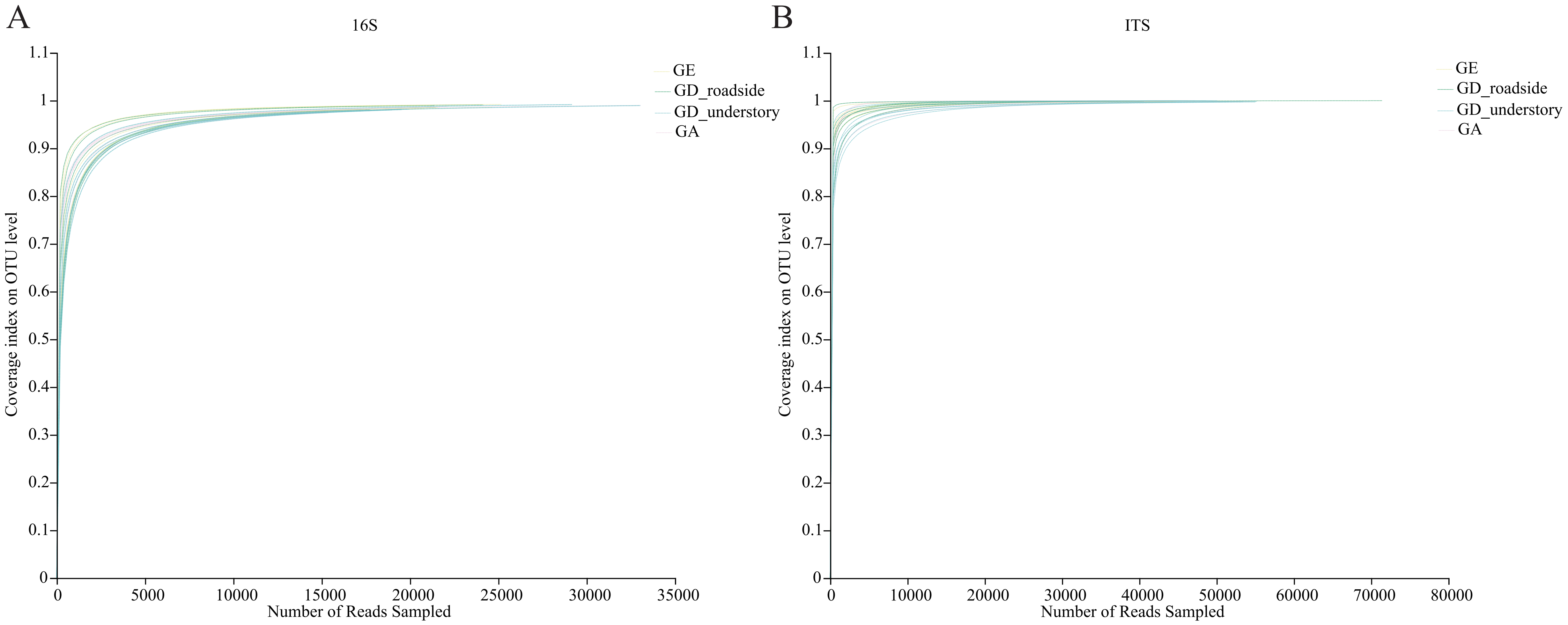

Supplementary Figure 1 An analysis of the rarefaction curve of 16S rRNA and ITS sequence. A: 16S rRNA sequence; B: ITS sequence. ITS, internal transcribed spacer.
