# Supplementary material for: The structure and diversity of bacteria and fungi in the roots and rhizosphere soil of three different species of Geodorum
Source: BMC Genomics. 2024 Feb 28;25:222. doi: 10.1186/s12864-024-10143-2 (PMC10903027; doi:10.1186/s12864-024-10143-2)
Supplement: Supplementary file 2 — Supplementary Material 2. [file 12864_2024_10143_MOESM2_ESM.pdf]

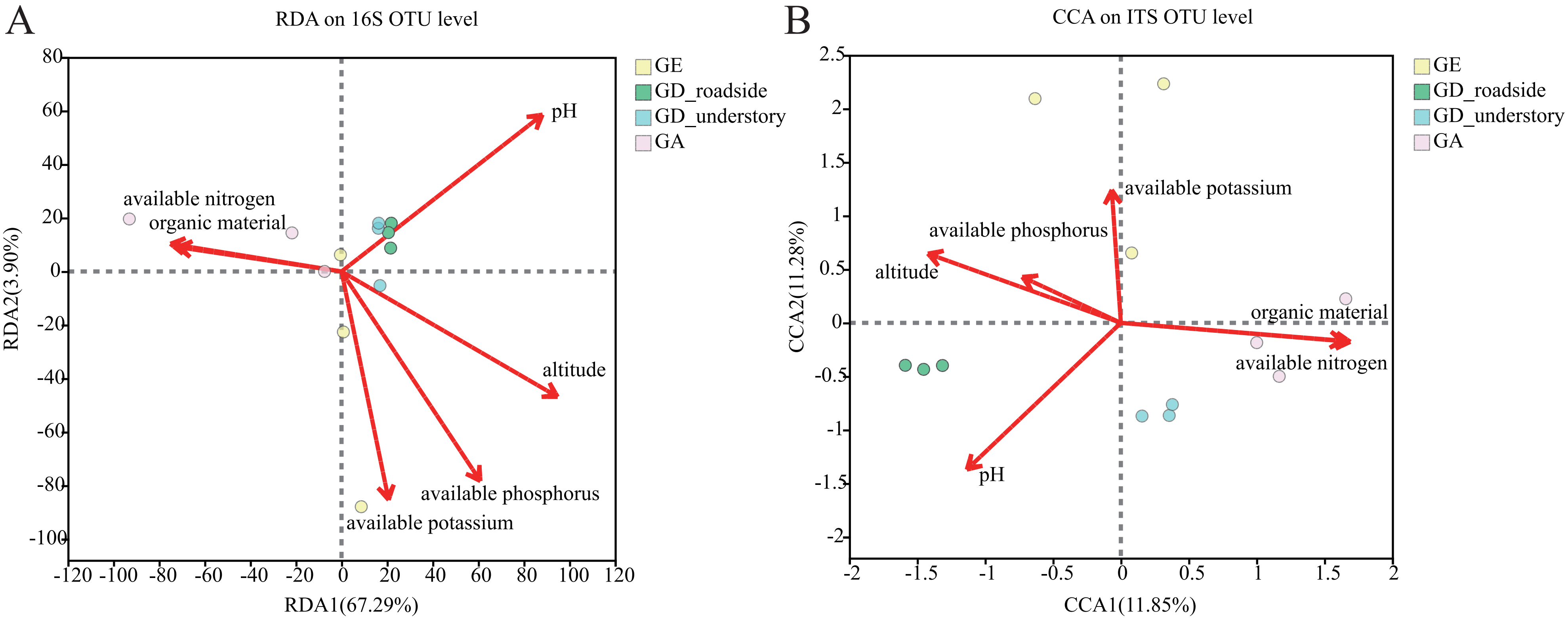

Supplementray Figure 2 Diagram of RDA and CCA analysis. A: RDA on 16S OTU level; B: CCA on ITS OTU level. The red arrow represents the quantitative environmental factor, and the length of the arrow of the environmental factor can represent the degree of influence of the environmental factor on the species data (the explanatory amount). The angles between the arrows of environmental factors represent positive and negative correlations (acute: positive correlation; Obtuse Angle: negative correlation; Right Angle: no correlation); The distance between the projection point and the origin represents the relative influence of environmental factors on the sample community distribution.
