# Supplementary figures and images for: The structure and diversity of bacteria and fungi in the roots and rhizosphere soil of three different species of Geodorum
Source: BMC Genomics. 2024 Feb 28;25:222. doi: 10.1186/s12864-024-10143-2 (PMC10903027; doi:10.1186/s12864-024-10143-2)

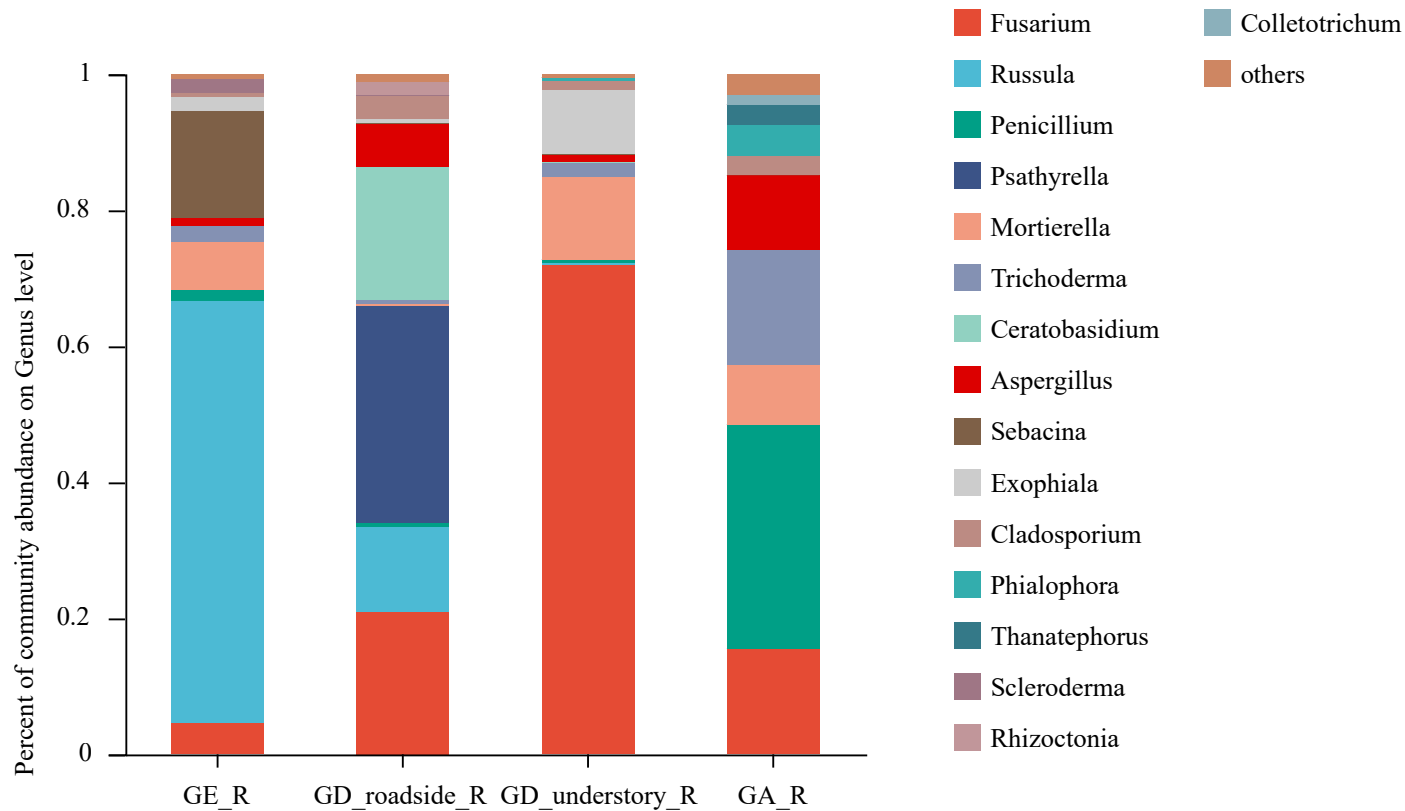

Supplementary Figure 3 The mycorrhizal fungal composition in the roots of *Geodorum*.

Supplement: Supplementary file 3 — Supplementary Material 3. [file 12864_2024_10143_MOESM3_ESM.pdf]
