# Supplementary material for: The structure and diversity of bacteria and fungi in the roots and rhizosphere soil of three different species of Geodorum
Source: BMC Genomics. 2024 Feb 28;25:222. doi: 10.1186/s12864-024-10143-2 (PMC10903027; doi:10.1186/s12864-024-10143-2)
Supplement: Supplementary file 4 — Supplementary Material 4. [file 12864_2024_10143_MOESM4_ESM.pdf]

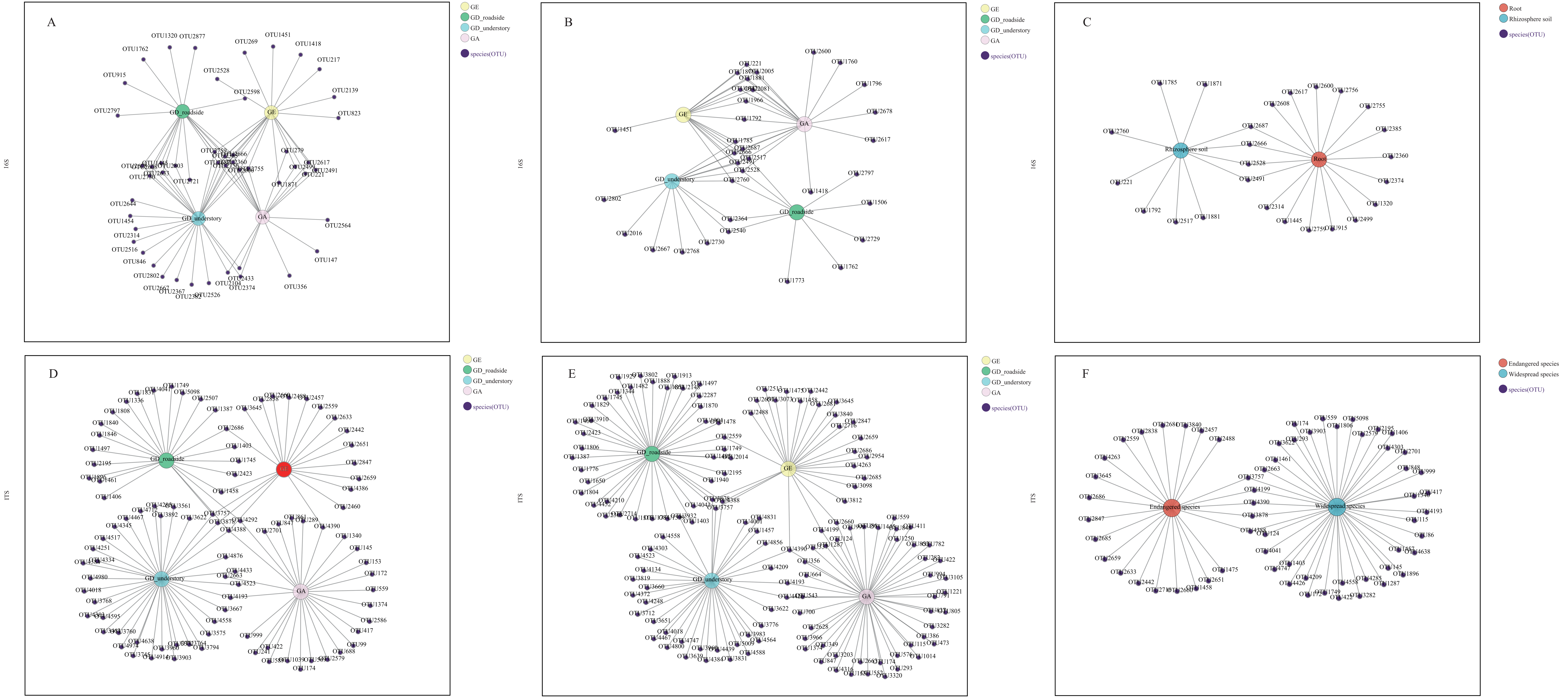

Supplementary Figure 4 Diagram of the co-occurrence network. A: in the roots between GA, GD_roadside, GD_understory, and GA groups at the 16S OTU level; B: in the rhizosphere soil between GA, GD_roadside, GD_understory, and GA groups at the 16S OTU level; C: between the root and rhizosphere soil groups at the 16S OTU level; D: in the roots between GA, GD_roadside, GD_understory, and GA groups at the ITS OTU level; E: in the rhizosphere soil between GA, GD_roadside, GD_understory, and GA groups at the ITS OTU level; F: between the endangered and widespread species groups at the ITS OTU level. The network contains the species node and sample node, and their wires represent that the sample contains the species. GA, *Geoderma attenuatum*; GD, *Geodorum densiflorum*; GE, *Geodorum eulophioides*; ITS, internal transcribed spacer; OTU, operational taxonomic unit.
