# Supplementary material for: The structure and diversity of bacteria and fungi in the roots and rhizosphere soil of three different species of Geodorum
Source: BMC Genomics. 2024 Feb 28;25:222. doi: 10.1186/s12864-024-10143-2 (PMC10903027; doi:10.1186/s12864-024-10143-2)
Supplement: Supplementary file 6 — Supplementary Material 6. [file 12864_2024_10143_MOESM6_ESM.pdf]

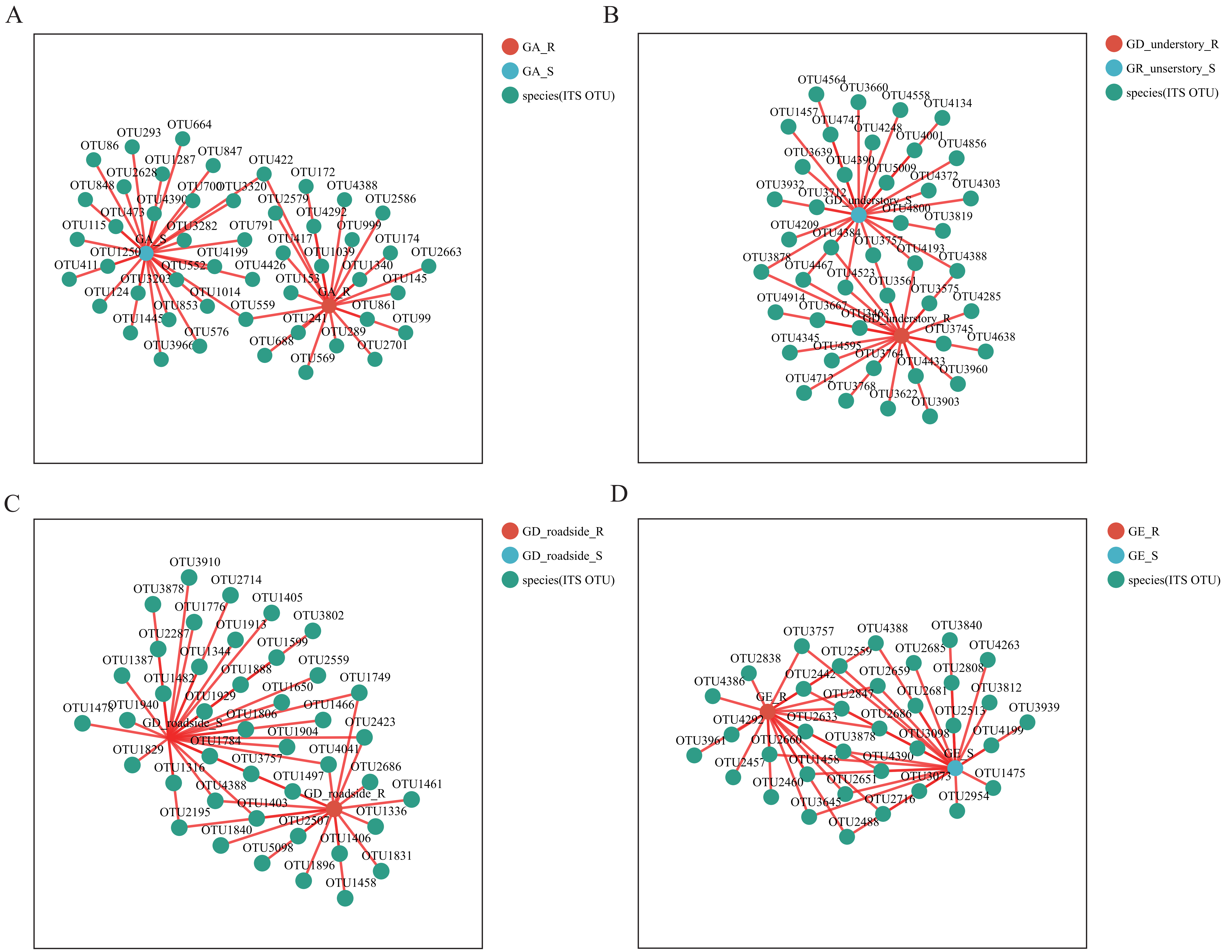


Supplementary Figure 6 Diagram of the co-occurrence network at the ITS OTU. A: in GA; B: in GD_understory; C: in GD_roadside; D: in GE. The network contains the species node and sample node, and their wires represent that the sample contains the species. GA, *Geoderma attenuatum*; GD, *Geodorum densiflorum*; GE, *Geodorum eulophioides*; ITS, internal transcribed spacer; OTU, operational taxonomic unit.
